# Spectral Power Analysis of Delta Waves in Neonatal Electroencephalography: A Tool for Assessing Brain Maturation and Injury

**DOI:** 10.7759/cureus.80680

**Published:** 2025-03-16

**Authors:** Yuma Kitase, Takehiko Hiroma, Yosuke Onishi, Yui Koyabu, Sora Jones, Ai Yoshino, Sora Hayashi, Haru Hayashi, Rin Hayashi, Seiya Shiraki, Chie Ishikawa, Yukihide Miyosawa, Dila Zafer, Atsuko Oba, Fumiya Yamaide, Kohei Kashima, Tadashi Shiohama, Katsunori Fujii, Tetsuo Kubota

**Affiliations:** 1 Department of Pediatrics, International University of Health and Welfare, Narita Hospital, Narita, JPN; 2 Division of Neonatology, Nagano Children's Hospital, Azumino, JPN; 3 Faculty of Medicine, International University of Health and Welfare, Narita Hospital, Narita, JPN; 4 Faculty of Medicine, Tomaya University, Toyama, JPN; 5 Faculty of Medicine, Nagoya City University, Nagoya, JPN; 6 Faculty of Dentistry, School of Dentistry, Asahi University, Mizuho, JPN; 7 Faculty of Medicine, Comenius University, Bratislava, SVK; 8 Department of Psychology, Aichi University of Education, Kariya, JPN; 9 Department of Pediatrics, Shinshu University Hospital, Matsumoto, JPN; 10 Department of Neurology, Basaksehir Cam and Sakura City Hospital, University of Health Sciences, Istanbul, TUR; 11 Department of Pediatrics, Anjo Kosei Hospital, Anjo, JPN

**Keywords:** brain injury evaluation, brain maturation, delta waves, neonatal eeg, spectral power analysis

## Abstract

Recent advances in neonatal care have improved survival rates of preterm infants but highlighted the persistent challenge of neurological impairments. This study focuses on delta wave analysis in neonatal electroencephalography (EEG) as a marker for brain maturation and injury. Using quantitative EEG methods, including spectral power analysis, we examined 399 EEG recordings from infants with gestational ages spanning 23-42 weeks. Results demonstrated significant maturation-related changes in delta wave spectral power across cortical regions, particularly in lower-frequency bands. Suppression of delta wave power correlated with visual assessments of brain injury severity. These findings suggest that delta wave spectral power analysis enhances the precision of brain function evaluation, providing a valuable complement to conventional methods such as amplitude-integrated EEG (aEEG). This approach holds potential for improving early diagnosis and guiding therapeutic interventions for neonatal brain injuries.

## Introduction

Recent advancements in neonatal and perinatal medicine have drastically improved the survival rates of preterm infants, even among those born at extremely early gestational ages [1,2]. These medical achievements, however, have not eliminated the prevalence of neurological complications. Perinatal brain injury remains a significant issue, with many survivors experiencing lifelong conditions such as cerebral palsy, cognitive impairments, epilepsy, and sensorimotor deficits [3-6]. Despite progress in experimental treatments, such as stem cell therapy for hypoxic-ischemic encephalopathy, effective strategies to mitigate or prevent these injuries are still in development [7-14].

Accurate and early evaluation of brain function is essential for identifying at-risk infants and initiating timely therapeutic interventions. Current clinical methods include head ultrasound, magnetic resonance imaging (MRI), and electroencephalography (EEG). Head ultrasound, though widely accessible, has limited sensitivity for functional assessments [15]. MRI provides comprehensive structural and functional insights but is often impractical for critically ill or extremely premature infants due to logistical challenges and instability risks [16]. By contrast, EEG, particularly amplitude-integrated EEG (aEEG), is the gold standard for bedside brain function evaluation [17-19]. However, aEEG predominantly relies on amplitude-based visual interpretation, which requires specialized training and may overlook nuanced changes in brain activity [20-22].

The rapid development of the fetal brain during the third trimester is reflected in weekly changes in neonatal EEG patterns, particularly in the amplitude and frequency of delta waves [23,24]. Delta waves, characterized by high amplitude and low frequency (0.5-4.0 Hz), are key indicators of brain maturation. Traditional methods of EEG analysis have focused on visual assessments of amplitude and sleep cycle patterns, which are effective but limited in sensitivity. To address these limitations, this study employs quantitative EEG (qEEG) methods, specifically spectral power analysis, to objectively examine frequency-related changes in delta waves across different gestational ages. This approach allows us to identify how delta wave suppression correlates with brain injury severity and provides insights into developmental trajectories.

By focusing on frequency rather than relying solely on amplitude, this study complements traditional aEEG methodologies. Delta wave spectral power analysis enables a more detailed understanding of brain maturation and injury mechanisms, offering a robust framework for improving neonatal brain function evaluation. This frequency-centric analysis has the potential to refine clinical assessments, facilitate early diagnosis, and guide therapeutic decisions, ultimately improving outcomes for preterm infants [25-28].

## Materials and methods

Study subjects

This retrospective study analyzed EEG recordings conducted as routine examinations between April 2010 and March 2011 at Nagano Children's Hospital. This study was approved by the Ethics Committee of Nagano Children's Hospital (Approval Number: S-06-119). A total of 430 hospitalized patients were included, comprising 286 preterm infants (<37 weeks of gestation) and 144 full-term infants (≥37 weeks of gestation). Thirty-one cases with EEG measurements taken under sedation were excluded based on exclusion criteria. The mean gestational age was 35 weeks two days ± two days (range: 23 weeks one day to 42 weeks five days), and the mean birth weight was 1735.0 ± 43.5 g.

Data acquisition

EEG recordings were performed using the Neurofax system (EEG-1214, Nihon Kohden, Tokyo, Japan) in accordance with the international 10-20 electrode placement system. EEG data were recorded using specific channels corresponding to predefined electrode placements. The channels included FP1-C3, C3-O1, FP2-C4, C4-O2, FP1-T3, T3-O1, FP2-T4, and T4-O2. These were mapped as follows: FP1-C3 was recorded using channels 1 to 5, C3-O1 using channels 5 to 9, FP2-C4 using channels 2 to 6, C4-O2 using channels 6 to 10, FP1-T3 using channels 1 to 15, T3-O1 using channels 15 to 9, FP2-T4 using channels 2 to 16, and T4-O2 using channels 16 to 10. The initial EEG recording was conducted once the patient’s vital signs stabilized after birth. Subsequent measurements were taken, whenever feasible, on postnatal days one, four, and seven, and then every two weeks until discharge. Each EEG session lasted approximately two hours and was conducted after feeding.

Data analysis

The recorded data were processed using the EEG analysis software Persyst Advanced Review (Persyst, Solana Beach, CA, USA). Data processing included the application of a time constant of 0.16 seconds, a low-pass filter set at 35 Hz, and notch filters applied at both 50 Hz and 60 Hz to minimize noise. For frequency analysis, the data were segmented into windows of four seconds, with a frequency resolution of 0.25 Hz. Delta waves are high-amplitude, low-frequency (0.5-4.0 Hz) waves that are prominently observed in neonatal EEGs. The characteristics of neonatal EEGs are based on the gradual increase in the frequency of slow waves as gestational age progresses. Specifically, the frequency is less than 1 Hz at 26 weeks or earlier postconception, approximately 1 Hz at 27-28 weeks, 1-1.5 Hz at 29-30 weeks, around 1.5 Hz at 31-32 weeks, 1.5-2 Hz at 33-34 weeks, around 2 Hz at 35-36 weeks, and greater than 2 Hz at 37-38 weeks. Delta waves were analyzed based on these frequency changes [29].

The EEGs were interpreted by experienced pediatric neurologists who assessed brain maturation based on the recordings. The degree of EEG suppression was classified according to previously reported methods. The grading of EEG activity suppression was classified as follows: Grade 1, minimal suppression, characterized by the flattening of the low-amplitude segments of the alternating trace pattern (TA), transitioning to a discontinuous tracing (D). In quiet sleep (QS), D is observed instead of TA. High-voltage slow pattern (HVS) is preserved or shows only a slight reduction. In active sleep (AS), the low-voltage irregular pattern (LVI) and mixed pattern (M) correspond to the sleep state, maintaining the relationship between sleep cycles and EEG patterns. Grade 2, mild suppression: HVS disappears, and LVI, M, and D become the predominant patterns. The interburst interval (IBI) in D remains within the normal range. In AS, a reduction in M or the appearance of D is observed, while LVI appears during QS. Grade 3, moderate suppression: M disappears, leaving only LVI and D. The IBI in D becomes prolonged. Although the distinction between active and QS is preserved, the biphasic cyclic nature of sleep is lost. Grade 4, marked suppression: The sleep cycle disappears, and only discontinuous EEG patterns remain. The IBI in D is significantly prolonged. Grade 5, maximal suppression: EEG recordings over extended periods exhibit only flat waveforms. Reduced amplitude was classified based on the maximum amplitude of delta waves as follows: Mildly reduced amplitude was defined as a maximum delta wave amplitude of ≤200 µV for a postmenstrual age of <30 weeks and ≤150 µV for a postmenstrual age of ≥30 weeks. Severely reduced amplitude was defined as a maximum delta wave amplitude of 20-50 µV. Flat EEG was defined as a maximum delta wave amplitude of ≤20 µV.

Statistical analyses

Data are represented as the mean ± standard error of the mean (SEM). Data were tested for normality with the Shapiro-Wilk Test. Parametric statistical differences between the two groups were established with a t-test, and non-parametric differences between the two groups were established with a Mann-Whitney U-test. For statistical analysis of three or more groups, parametric tests were not conducted due to the non-normal distribution of the data. Instead, for non-parametric data with three or more groups, the Kruskal-Wallis test was employed. GraphPad Prism 9.3.1 software was used for statistical analysis.

## Results

Assessment of maturity

**Figure 1:**
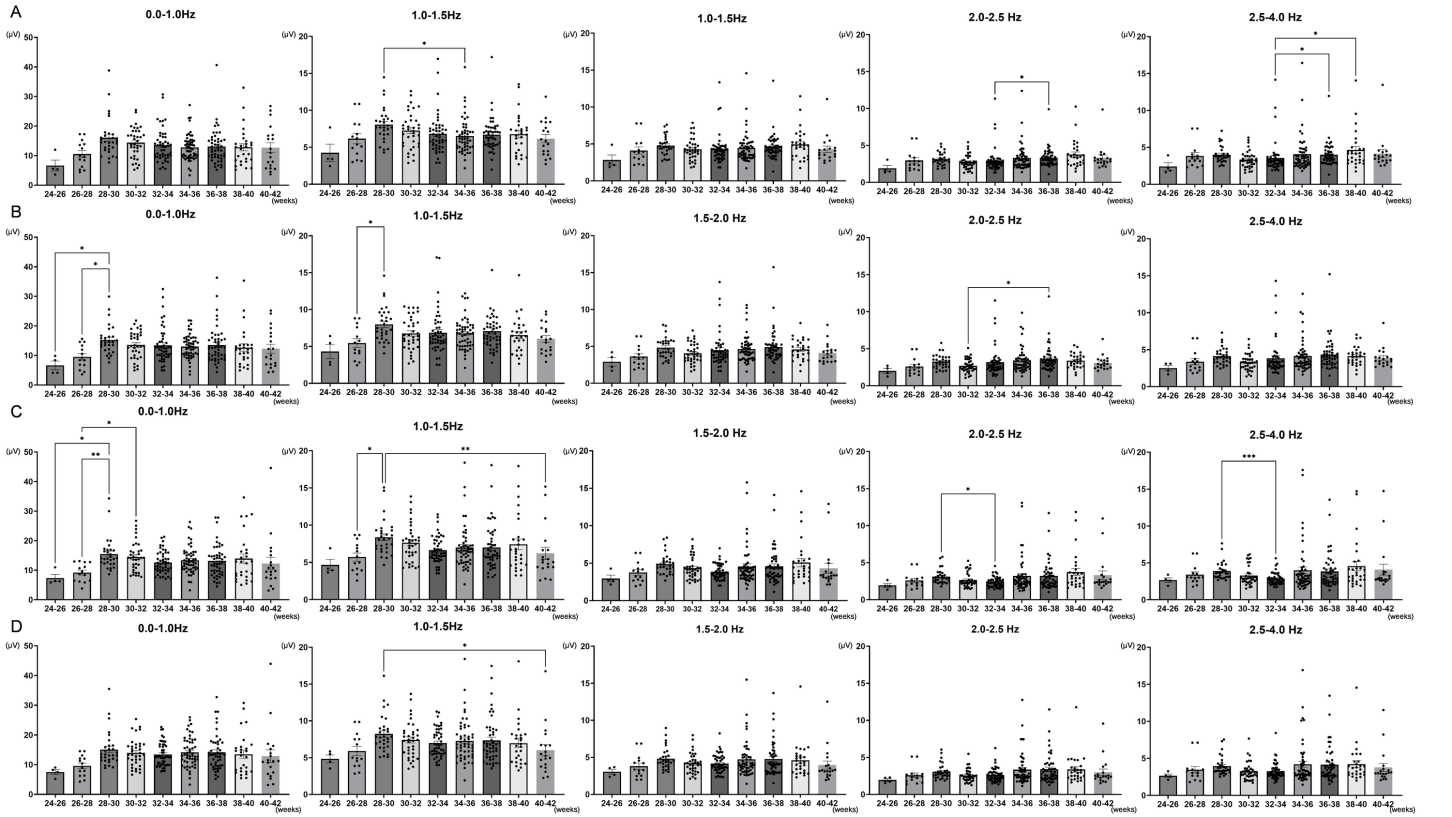
Differences in spectral power by measurement sites throughout development The changes in the spectral power of delta waves corresponding to gestational age were analyzed across four regions: anterior frontal (AF)-central (C) (Panel A), AF-temporal (T) (Panel B), C-Occipital (O) (Panel C), and T-O (Panel D). Between 24 and 32 weeks, a significant increase in spectral power at 0.0-1.0 Hz was observed in AF-T and C-O regions (Panels B, C). In addition, relatively higher frequencies at 2.0-4.0 Hz in the AF-C region showed a significant increasing trend (A). Normality was assessed using the Shapiro-Wilk Test, and statistical differences between groups were determined by the Kruskal-Wallis test. (*: p < 0.05; **: p < 0.01; ***: p < 0.001)

Delta waves showed significant changes in the AF-T and C-O regions between 24 and 30 weeks of postmenstrual age. The changes in the spectral power of delta waves corresponding to gestational age were analyzed across four regions: Anterior frontal (AF)-central (C), AF-temporal (T), C-occipital (O), and T-O. The results showed a gradual increase in the spectral power of low-frequency delta waves (0.0-1.0 Hz) in the AF-T and C-O regions between 24 and 30 weeks (AF-T: 24-26 weeks 6.6 ± 1.47 vs. 26-28 weeks 9.5 ± 1.07, p < 0.05; 24-26 weeks 6.6 ± 1.47 vs. 28-30 weeks 15.2 ± 0.89, p < 0.05; C-O: 24-26 weeks 7.4 ± 1.21 vs. 28-30 weeks 15.5 ± 1.02, p < 0.05; 26-28 weeks 9.2 ± 0.83 vs. 28-30 weeks 15.5 ± 1.02, p < 0.01; 26-28 weeks 9.2 ± 0.83 vs. 30-32 weeks 14.5 ± 0.84, p < 0.05, Figures 1A, 1C). Between 28 and 34 weeks, a decrease in spectral power within the 2.0-4.0 Hz range was observed in the C-O region (2.0-2.5 Hz: 28-30 weeks 3.11 ± 0.18 vs. 32-34 weeks 2.38 ± 0.10, p < 0.05; 2.5-4.0 Hz: 28-30 weeks 3.9 ± 0.22 vs. 32-34 weeks 2.9 ± 0.12, p < 0.001, Figure 1C). From 32 to 40 weeks, the spectral power in the relatively higher-frequency range of 2.0-4.0 Hz showed a significant increasing trend in the AF-C region (32-34 weeks 3.6 ± 0.32 vs. 36-38 weeks 4.0 ± 0.22, p < 0.05; 32-34 weeks 3.6 ± 0.32 vs. 38-40 weeks 4.6 ± 0.48, p < 0.05, Figure 1A).

**Figure 2:**
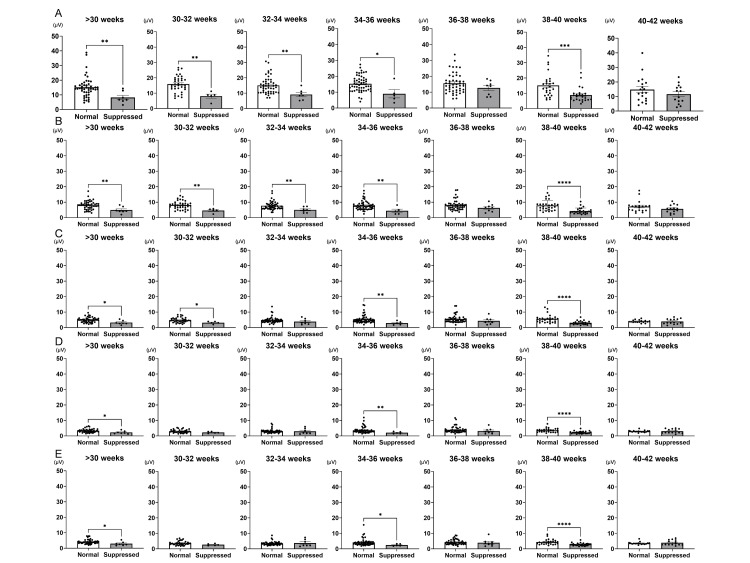
Differences in the detection of EEG suppression by frequency across gestational age EEG: electroencephalography Focusing on delta wave frequencies, the comparison of spectral power across the entire brain between the control and suppression groups was conducted for each frequency band at different gestational ages. Panel A shows 0.0-1.0 Hz, Panel B shows 1.0-1.5 Hz, Panel C shows 1.5-2.0 Hz, Panel D shows 2.0-2.5 Hz, and Panel E shows 2.5-4.0 Hz. Lower frequencies tended to show more significant differences between normal EEG and suppressed EEG depending on gestational age. Normality was assessed using the Shapiro-Wilk Test. Parametric statistical differences between the two groups were established with a t-test, and non-parametric differences between the two groups were established with a Mann-Whitney U-test. (*: p < 0.05; **: p < 0.01; ***: p < 0.001; ****: p < 0.0001)

The spectral power of delta waves significantly decreases due to suppression. Next, a comparison of the spectral power across the entire brain, focusing on delta wave frequencies, between the normal group and the suppressed group was conducted for each frequency across different gestational ages. At each gestational age, a more significant reduction in spectral power was observed at lower frequencies. At 0.0-1.0 Hz, significant suppression was observed at all weeks except for 36-38 weeks and beyond 40 weeks (>30 weeks normal, N: 15.20 ± 1.01, n = 48, vs. suppression, D: 8.20 ± 1.51, n = 7, p < 0.01; 30-32 weeks N: 15.96 ± 0.87, n = 36 vs. D: 8.07 ± 1.30, n = 5, p < 0.01; 32-34 weeks N: 15.06 ± 0.80, n = 48 vs. D: 9.11 ± 1.28, n = 7, p < 0.01; 34-36 weeks N: 15.28 ± 0.69, n = 53 vs. D: 9.01 ± 2.60, n = 5, p < 0.05; 36-38 weeks N: 15.63 ± 0.90, n = 48 vs. D: 12.68 ± 1.61, n = 8, n.s.; 38-40 weeks N: 15.11 ± 1.45, n = 27 vs. D: 8.84 ± 0.91, n = 26, p < 0.01; 40-42 weeks N: 14.78 ± 2.03, n = 19 vs. D: 11.62 ± 1.74, n = 14, n.s., Figure 2A). However, when the delta wave frequency exceeded 2.5 Hz, significant differences were only observed at gestational ages <30 weeks, 34-36 weeks, and 38-40 weeks (>30 weeks N: 3.30 ± 0.16, n = 47 vs. D: 2.27 ± 0.37, n = 7, p < 0.05; 30-32 weeks N: 3.02 ± 0.17, n = 36 vs. D: 2.25 ± 0.24, n = 5, n.s.; 32-34 weeks N: 3.08 ± 0.18, n = 47 vs. D: 2.98 ± 0.70, n = 7, n.s., 34-36 weeks N: 3.69 ± 0.28, n = 52 vs. D: 2.08 ± 0.30, n = 5, p < 0.05; 36-38 weeks N: 3.96 ± 0.29, n = 48 vs. D: 3.20 ± 0.66, n = 8, n.s.; 38-40 weeks N: 3.65 ± 0.27, n = 25 vs. D: 2.27 ± 0.15, n = 26, p < 0.0001; 40-42 weeks N: 2.98 ± 0.16, n = 17 vs. D: 3.07 ± 0.36, n = 14, n.s., Figure 2D). These findings suggest that delta waves with lower frequencies may be more useful for assessing suppression.

**Figure 3:**
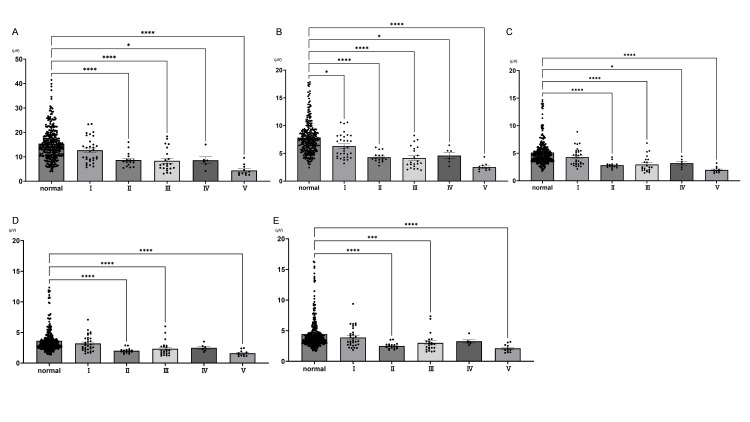
Comparison of EEG suppression grade classification by frequency with normal EEG EEG: electroencephalography The spectral power was analyzed for all neonates according to the degree of EEG activity suppression and normal levels. Panel A shows 0.0-1.0 Hz, Panel B shows 1.0-1.5 Hz, Panel C shows 1.5-2.0 Hz, Panel D shows 2.0-2.5 Hz, and Panel E shows 2.5-4.0 Hz. Significant differences were observed at 1.0-1.5 Hz between normal EEG and all suppression grades. Additionally, lower frequencies tended to show more significant differences compared to normal EEG. Normality was assessed using the Shapiro-Wilk test, and statistical differences between groups were determined by the Kruskal-Wallis test. (*: p < 0.05; **: p < 0.01; ***: p < 0.001; ****: p < 0.0001)

The spectral power decreases according to the grade classification of suppression. Next, the spectral power was analyzed for all neonates according to the degree of EEG activity suppression and normal levels. Compared to the normal group, mild suppression (Grade 1) showed a significant decrease in spectral power at all frequencies in the 1.0-1.5 Hz range (normal: 7.83 ± 0.17, n = 304; Grade 1: 6.32 ± 0.39, n = 31, p < 0.05; Grade 2: 4.29 ± 0.23, n = 16, p < 0.0001; Grade 3: 4.16 ± 0.41, n = 22, p < 0.0001; Grade 4 4.59 ± 0.58, n = 6, p > 0.05; Grade 5: 2.52 ± 0.22, n = 11, p < 0.0001, Figure 3), with the difference being more clearly seen at the lower frequencies.

## Discussion

This study analyzed approximately 400 neonatal EEGs from 23 to 42 weeks of gestational age using spectral power analysis. It successfully captured developmental changes in delta waves across different cortical regions based on subdivided delta wave patterns. Furthermore, the evaluation of EEG activity suppression due to brain injury demonstrated that spectral power analysis sensitively detected this suppression, with specific frequencies showing more pronounced suppression. These findings suggest that spectral power analysis could serve as a valuable adjunctive tool for evaluating brain activity via EEG and for determining the suitability of therapeutic hypothermia in clinical practice.

The development of EEG patterns accompanying brain maturation during neonatal development is remarkable, with delta waves exhibiting changes by gestational age [29]. Kato et al. previously reported the utility of amplitude spectral analysis in evaluating the maturation-related changes in delta activity in preterm infants. Their study analyzed 10 preterm infants between 29 and 34 weeks of gestational age, finding pronounced changes in the occipital region and frequencies below 1 Hz [30]. Similarly, Jennekens et al. conducted spectral analysis of EEGs in preterm infants aged 28-36 weeks, reporting that maturation-related changes in spectral parameters were primarily observed in the frontal and temporal lobes [31]. In our analysis, we observed distinctive maturation-related changes in the AF-T and C-O regions, with significant alterations noted at frequencies below 1 Hz. Consistent with previous studies, we also captured specific regional changes in delta waves below 1 Hz [30]. These findings underscore the significance of focusing on prominent changes in delta waves for advancing neonatal EEG analysis, particularly in understanding the developmental trajectories of the neonatal brain. It has been reported that the development of the frontal lobe during the third trimester of pregnancy is remarkable, accompanied by significant morphological changes. Active folding of the frontal lobe is observed between 26 and 28 weeks of gestation, with substantial changes detected in the middle frontal gyrus, superior frontal gyrus, frontal pole, and orbitofrontal gyrus. By 30 to 32 weeks of gestation, most of the primary sulci in the frontal lobe become distinguishable [32,33]. In our study, spectral power analysis successfully captured changes in frontal lobe activity, reflecting the rapid development of the frontal lobe. Since the development of the frontal lobe during this period is considered critically important for the emergence of higher cognitive functions [33], the observed changes in delta wave spectral power suggest the potential for predicting future developmental trajectories.

Our results also revealed that the lower the frequency of delta waves, the more significant the reduction in spectral power observed in EEG suppression across gestational weeks. Visual assessment of EEG suppression often relies on reductions in delta wave amplitude [34], which may overlook subtle changes in other clinically relevant frequency bands. Consequently, relying solely on delta wave amplitude reduction could lead to an underestimation of EEG suppression [35]. Based on our findings, a more granular analysis of frequencies, specifically focusing on lower-frequency delta waves, may provide a more sensitive and accurate evaluation of EEG suppression.

Our study also demonstrated a significant correlation between visually assessed EEG suppression and spectral power. HIE is a severe brain injury occurring during the neonatal period, with an incidence of 1.5-2.5 per 1,000 live births in developed countries [36] and 2.3-30.6 per 1,000 in developing countries [37]. HIE is a condition associated with lifelong neurological impairments, including cerebral palsy, epilepsy, cognitive and intellectual disabilities, developmental delays, and visual and auditory deficits [38,39]. The standard treatment for HIE is therapeutic hypothermia, and neonatal EEG, particularly aEEG, is highly valuable for initiating this therapy in affected neonates [40]. aEEG is effective in assessing moderate to severe encephalopathy and is utilized as a criterion for starting therapeutic hypothermia [40]. Early aEEG traces have been reported to demonstrate high prognostic accuracy within the first six hours after birth [41]. Additionally, aEEG provides objective information about brain function, even in cases where physical examinations may be challenging or subjective, making it an indispensable tool for evaluating neonatal brain activity [40]. As such, aEEG and EEG provide valuable information for assessing brain function. However, it is crucial to note that these tools should be used in conjunction with other clinical and physiological indicators, such as evidence of perinatal hypoxic-ischemic events and the presence of significant neonatal encephalopathy in clinical evaluations. This comprehensive approach ensures the accurate determination of the appropriateness of therapeutic hypothermia [42]. In cases of HIE, the limited time window for initiating therapeutic hypothermia underscores the importance of rapid and accurate assessment. While standard EEG and aEEG, presented as compressed traces, require specific training to interpret, incorporating additional indicators of brain activity can provide more robust evidence. If the spectral power analysis we conducted can complement these methods, it could significantly enhance the accuracy of decision-making and evaluation in such critical scenarios.

In this study, we were able to analyze a substantial cohort of 399 cases spanning a wide gestational age range of 25 to 42 weeks. However, a limitation of our study is that we did not link the spectral power analysis data with the developmental outcomes of the patients. As a result, we could not examine how the spectral power findings correspond to specific developmental phenotypes. Additionally, previous studies have reported associations between alpha and beta waves and future developmental trajectories [43]. Therefore, understanding the relationship between the characteristics of these subdivided delta waves and developmental outcomes remains an important subject for future research.

## Conclusions

In conclusion, EEG testing, which allows for real-time bedside measurement of brain function in preterm and term infants at risk of brain injury, is considered the gold standard and a critical determinant for initiating therapeutic hypothermia. It is also a highly valuable tool for providing reliable information regarding future developmental outcomes. Spectral power analysis, which objectively evaluates brain function during the dramatic changes occurring in the brain during the third trimester of pregnancy, has proven to be extremely useful. Our findings indicate that spectral power analysis can provide strong and supplementary information for brain function evaluation. In the future, with a multifaceted approach to EEG analysis, this method could become a powerful tool for obtaining more refined and accurate data on brain function, which remains an area of considerable unknowns.
